# Development and Comparative Analysis of Electrochemically Etched Tungsten Tips for Quartz Tuning Fork Sensor

**DOI:** 10.3390/mi12030286

**Published:** 2021-03-08

**Authors:** Ashfaq Ali, Naveed Ullah, Asim Ahmad Riaz, Muhammad Zeeshan Zahir, Zuhaib Ali Khan, S. Shaukat Ali Shah, Muftooh Ur Rehman Siddiqi, Muhammad Tahir Hassan

**Affiliations:** 1 Department of Mechanical Engineering, University of Engineering & Technology, Peshawar 25000, Pakistan; me.ashfaqali@gmail.com (A.A.); zeeshan.zahir@uetpeshawar.edu.pk (M.Z.Z.); engr_zuhaib@uetpeshawar.edu.pk (Z.A.K.); calmshah@gmail.com (S.S.A.S.); 2 Department of Mechanical Engineering, CECOS University of IT and Emerging Sciences, Peshawar 25000, Pakistan; 3 Department of Mechanical Engineering, Bahauddin Zakariya University, Multan 60011, Pakistan; tahirqureshi@bzu.edu.pk

**Keywords:** scanning probe microscopy, tungsten tips, electrochemical etching, scanning electron microscope, quartz tuning fork sensor

## Abstract

Quartz Tuning Fork (QTF) based sensors are used for Scanning Probe Microscopes (SPM), in particular for near-field scanning optical microscopy. Highly sharp Tungsten (W) tips with larger cone angles and less tip diameter are critical for SPM instead of platinum and iridium (Pt/Ir) tips due to their high-quality factor, conductivity, mechanical stability, durability and production at low cost. Tungsten is chosen for its ease of electrochemical etching, yielding high-aspect ratio, sharp tips with tens of nanometer end diameters, while using simple etching circuits and basic electrolyte chemistry. Moreover, the resolution of the SPM images is observed to be associated with the cone angle of the SPM tip, therefore Atomic-Resolution Imaging is obtained with greater cone angles. Here, the goal is to chemically etch W to the smallest possible tip apex diameters. Tips with greater cone angles are produced by the custom etching procedures, which have proved superior in producing high quality tips. Though various methods are developed for the electrochemical etching of W wire, with a range of applications from scanning tunneling microscopy (SPM) to electron sources of scanning electron microscopes, but the basic chemical etching methods need to be optimized for reproducibility, controlling cone angle and tip sharpness that causes problems for the end users. In this research work, comprehensive experiments are carried out for the production of tips from 0.4 mm tungsten wire by three different electrochemical etching techniques, that is, Alternating Current (AC) etching, Meniscus etching and Direct Current (DC) etching. Consequently, sharp and high cone angle tips are obtained with required properties where the results of the W etching are analyzed, with optical microscope, and then with field emission scanning electron microscopy (FE-SEM). Similarly, effects of varying applied voltages and concentration of NaOH solution with comparison among the produced tips are investigated by measuring their cone angle and tip diameter. Moreover, oxidation and impurities, that is, removal of contamination and etching parameters are also studied in this research work. A method has been tested to minimize the oxidation on the surface and the tips were characterized with scanning electron microscope (SEM).

## 1. Introduction

Scanning Probe Microscopy (SPM) is a branch of imaging technique that scans the surface with the varying sharp probe whose diameter vary from size of the atom up to 10 nm [1]. Classification of the SPM technique can be broadly divided into two categories which are Scanning Tunneling Microscopy (STM) and Atomic Force Microscopy (AFM) [2]. In STM, a tunneling current between atomically sharp probes is used for scanning the surface. STM consists of small sharp conducting tip that scans across the sample [3]. In AFM, 100–200 micrometer cantilever with a sharp silicon nitride or silicon crystal tip is used for the scanning of non-conducting samples, that is, polymers and biological specimens with atomic resolution [4].

The SPM tip plays the role of the top electrode, while the bottom electrode is provided by an atomically flat metallic surface [5]. The clearness of the SPM images was observed to be associated with the cone angle of the SPM tip, with greater cone angles reliably creating atomically resolved images. Larger cone angle tips are more likely to provide better STM images, as a result of their greater mechanical stability and sharpness. Cone angle can be measured by taking optical images. The custom etching procedure allows one to create larger cone angles and consequently proved superior in reliably producing high-quality tips. This shows an ideal atomically sharp yet stable tip having a cone angle of about 60°. In general, tips with a cone angle of ~15° or greater gave excellent STM images and cone angles around 20° were more typical [6,7]. Optimum production of SPM tips is an important task which is vital for SPM techniques. So different methods are currently used for the production of sharp tips in which the etching technique is commonly used as a quick, inexpensive, appropriate and consistent method and also as a pre-treatment step for more precise production [8].

In the etching technique, the drop-off method is currently the most common and effective method used for the production of sharp tips [1]. Electrochemical etching basically results from the anodic dissolution of tungsten wire in an aqueous potassium hydroxide (KOH) or sodium hydroxide (NaOH) electrolyte solution in the ‘drop-off’ method. The typical method includes dipping of tungsten wire into potassium hydroxide (KOH) or sodium hydroxide (NaOH) electrolyte solution [5]. When the tip is biased, so etching begins at the air/electrolyte interface and continues into the dipped/immersed tungsten wire. Similarly, in the electrochemical process, tungsten wire is connected with the positive voltage through which etching takes place within the meniscus on the wire surface and below the marginal air/electrolyte interface [9]. So tungsten wire is oxidized to form tungstate anions at a node in the electrochemical reaction, which are soluble in water and hence the wire is partially dissolved. Likewise, when tungsten wire is submerged in the NaOH concentration solution, so the surface tension and capillary forces results in the creation of a solution meniscus around the tip wire [10]. In defining the final form and shape of the tip, the meniscus shape plays a significant part of the etching reaction at the bottom acts faster than at the top of meniscus. Moreover OH- ion concentration is also lower at the top of the meniscus than in the total solution [11]. Thus, at the top of the meniscus, the etching process takes place at a slower pace. So, tungsten anions are produced where etching takes place at a higher rate, which will flow downwards. Therefore, a necking effect is found in the meniscus where the etching rate is increased. At some level, this portion of the wire becomes so thin that its lower end weight cannot be sustained due to less tensile strength, so the latter drops off and a sharp tip is remained. When the tips drop off, the etching voltage needed to be switched off as quickly as possible for producing sharp tips and avoiding blunt and large diameter tips. In conclusion, the remaining upper portion is used as the end product, that is, tungsten tip and in some cases both the portions, that is, upper and lower are used as tungsten tips, increasing the chances of double tips due to the formation of double meniscus [5]. The above-mentioned method is known as the “drop-off” method [1,5].

Looking to the literature, huge numbers of methods are used for the production of sharp tungsten tips [1]. In this work we decided to do a comparative study of AC etching, Meniscus etching and our proposed technique, that is, DC etching [12]. Moreover, a DC circuit is incorporated which will automatically switch off the current when just etching completes, so as to prevent tips from over etching and blunting. All these three methods are simple, economical, fast and manageable to produce tips with less tip diameter and large cone angles. Moreover, scanning electron microscope (SEM) and Optical Microscopic (OM) analysis confirmed the high success rate of the sharp tips [13].

## 2. Experimental Methods and Procedures

### 2.1. Alternating Current (AC) Etching

**Scheme 1:**
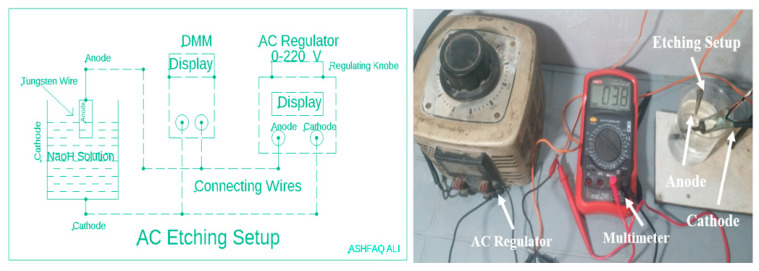
Schematic and Actual experimental setup of Alternating Current (AC) Etching Setup.

The first experimental method utilized for production of tungsten tips is electrochemical etching through Alternating Current voltage which is called AC etching. Performing electrochemical etching by using AC voltage yields in abundant development of H_2_ gas bubbles nearby cathode moving towards specimen, that is, tungsten tips. So microscopically irregular and tips with longer taper length are produced, which are not ideal for SPM use. In this process, the higher etching voltage causes a faster chemical reaction, thus producing [14] more H_2_ gas bubbles around the tungsten wire [11]. Some of these bubbles combine and grow larger with the passage of time until breaking. Due to this breakage of the bubbles, instability is produced in the solution, which yields less surface quality tips [6,15]. Moreover, for experimental purposes through AC etching, we have taken a regulator converting AC voltage from range of 0–220 V and used this voltage by adjusting with a multi-meter for experimental work. Instead, the effects of voltage and the concentration of solution [16] are checked using a variable voltage of 1–15 V taken from the AC regulator whose results are shown in below Tables 1 and 2. Finally experimental setup with a schematic diagram are shown below in Scheme 1.

### 2.2. Meniscus Etching

**Scheme 2:**
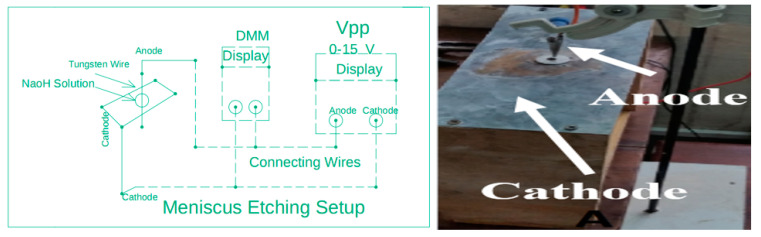
Schematic and actual experimental setup of Meniscus Etching Setup.

The second method on which we have performed experiments for the production of tungsten tips is the meniscus etching. Meniscus etching is a process in which tungsten wire is passed from the meniscus etching setup as we have shown in our proposed meniscus etching setup below in Scheme 2. Our proposed meniscus etching setup is a sheet of metal that has a hole of small diameter representing a ring like structure, which supports NaOH solution and forms the meniscus [17]. When the meniscus breaks up so the NaOH solution is continuously poured into the ring like structure through which the tungsten wire is passed. This setup requires less amount of NaOH solution as compare to electrochemical etching setup used in above AC etching. The Tungsten wire is given a positive charge, that is, connected with the anode and the sheet of metal having a hole of small diameter sustaining meniscus is cathode in this process. In this process, two sharp tips produced at the same time due to the formation of the double meniscus as is clear from the literature review [6]. Moreover, for each experiment, two samples are produced, both having the same applying voltage and ampere drawn from the supply but having different cone angle and tip diameter as shown in Table 3. This process also has some drawbacks; it takes more time due to the continuous breakage of the meniscus and also more chances of blunting the tip. As meniscus etching is a slow process due to the lower concentration of the meniscus solution and the continuous breakage of the meniscus around the tungsten wire, so at a lower voltage it takes more time for the completion of reaction. Therefore, we have started experimental work from a little high voltage to complete the reaction at an average time and to produce sharp tips.

### 2.3. Direct Current (DC) Etching

**Scheme 3:**
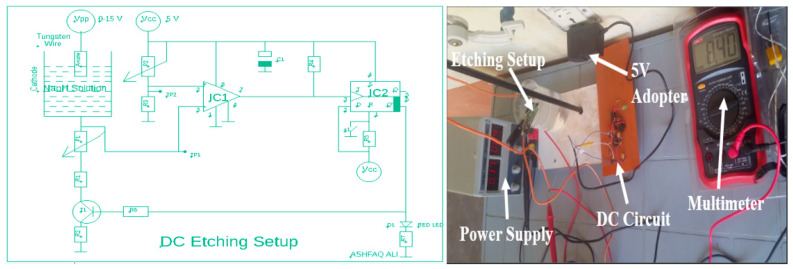
Schematic and actual experimental setup of DC Etching Setup.

The third method on which we have performed an experiment for the production of tungsten tips is DC etching. Electrochemical etching with a direct current is also performed for better results, which is called DC etching as shown below in Scheme 3. In DC etching, direct current is used for etching purposes. Here in DC etching, we have also incorporated a DC circuit which is a complex circuit used in order to channel the DC for etching [18]. The DC circuit will automatically switch up the current when etching is complete [19]. In this process, at lower DC voltages sharp tips with larger cone angles are produced due to the formation of strong necking effects, which are appropriate for SPM use [11]. Moreover, as a higher reaction rate quickly etches the lower part of the wire before the creation of a significant neck, so with higher DC voltages the drop-off is somewhat unlikely. Similarly, a positive DC etching voltage also eliminates disruptive gas formation at the etching site [6]. For our experimental purposes, using DC etching, we have taken a power supply working like a step down transformer converting 220 V into range of 1–15 V and used it for experimental work.

## 3. Results and Discussion

To produce SPM tips, there are various techniques and these techniques for the creation of very sharp tips [20]. However, the optimization of the process parameters for reproducibility and tip form/shape regulation is a concern. The latest attempts have come in the form of a basic electrochemical “drop-off” process to optimize tip manufacturing [21]. In this work, we have produced tips on three different electrochemical etching techniques which are AC, Meniscus and DC etching methods and also have produced some specific samples on each method as shown below in the experimental results. Highly sharp tips with controllable cone angles and tip diameter are provided by these simple “drop-off” methods. As is obvious from the literature, excellent SPM images were given by tips with a cone angle of ~15° or greater and a tip diameter up to 50 nm [6]. So, tip diameter and cone angles of all samples were checked and our results show that about ~70% of tips were produced having a cone angle greater than 20° and tip diameter less than 60 nm as shown in the below experimental data. In addition, etching voltage was tuned to be high enough to cause drop-off, but not too high to create tip apex blobbing. For all the experimental work performed, we kept some specific values of etching voltage [14] and concentration of solution, that is, 2 M and 4 M to show its effects on different etching procedures. Some of the important experimental results for 0.4 mm tips showing tip diameter, etching time and cone angle in 2 M and 4 M NaOH solution on AC, Meniscus and DC Etching, which we explored from our experimental work, are given in the subsections.

### 3.1. AC Etching Results

**Figure 1:**
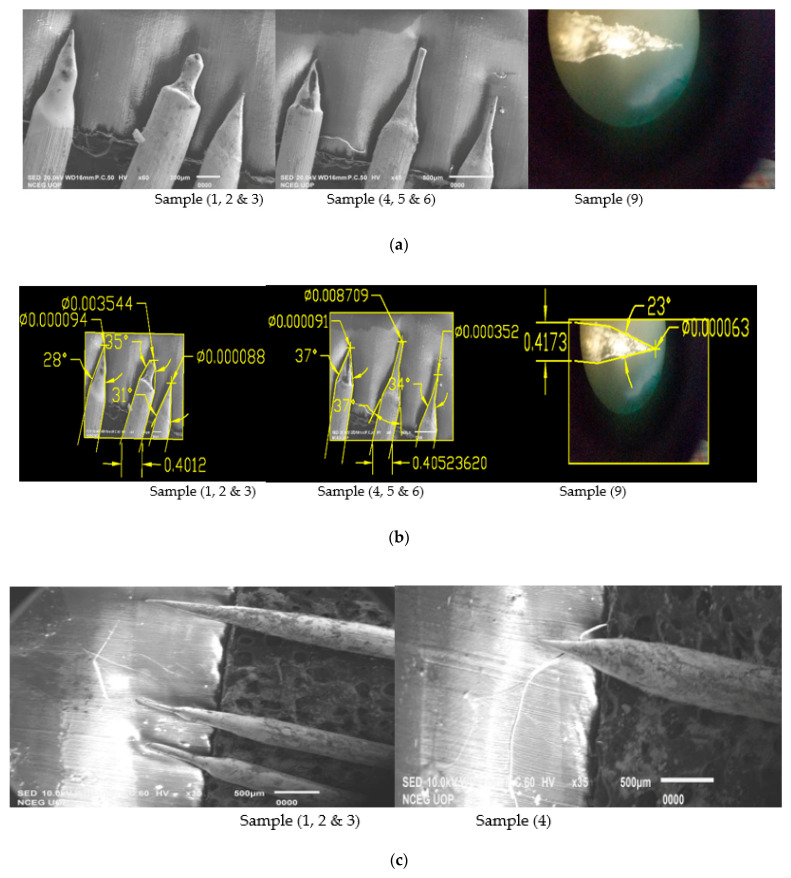

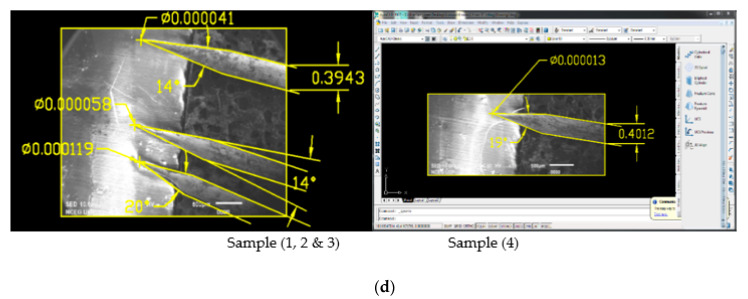
(**a**) Scanning electron microscope (SEM) and Optical Microscope (OM) Images of 0.4 mm tungsten tips prepared in 2 Molar NaOH solution (16 g/200 mL); (**b**) SEM and OM Images of 0.4 mm tungsten tips in 2 molar NaOH solution showing cone angle and tip diameter measured using AutoCAD Software; (**c**) SEM Images of 0.4 mm tungsten tips prepared in 4 Molar NaOH solution (32 g/200 mL); (**d**) SEM Images of 0.4 mm tungsten tips in 4 molar NaOH solution showing cone angle and tip diameter measured using AutoCAD Software.

On AC etching, 0.4 mm equivalent to 0.016-inch tungsten wire is dipped into 2 and 4 molar NaOH solution containing an electrode and applying a variable voltage of 1–15 V to show the effects of voltage and concentration of solution [16]. The process dissolved the wire partially at the air solution boundary line up to the formation of a sharp tip when the etching voltage is applied as shown in Figure 1c. In 2 molar solution, 16 g of NaOH is dissolved in 200 mL of distilled water while in 4 molar solution 32 g of NaOH is dissolved. Similarly, we have applied the etching voltage in such a way that we have connected tungsten wire to anode and the electrode submerged in the solution is connected with cathode in this process. Moreover, the cone angle and tip diameter of the produced tips are found by using AutoCAD Software whose details are shown in Figure 1. Experimental results and SEM images for the produced tips are shown in Table 1 and Figure 1.

**Figure 2:**
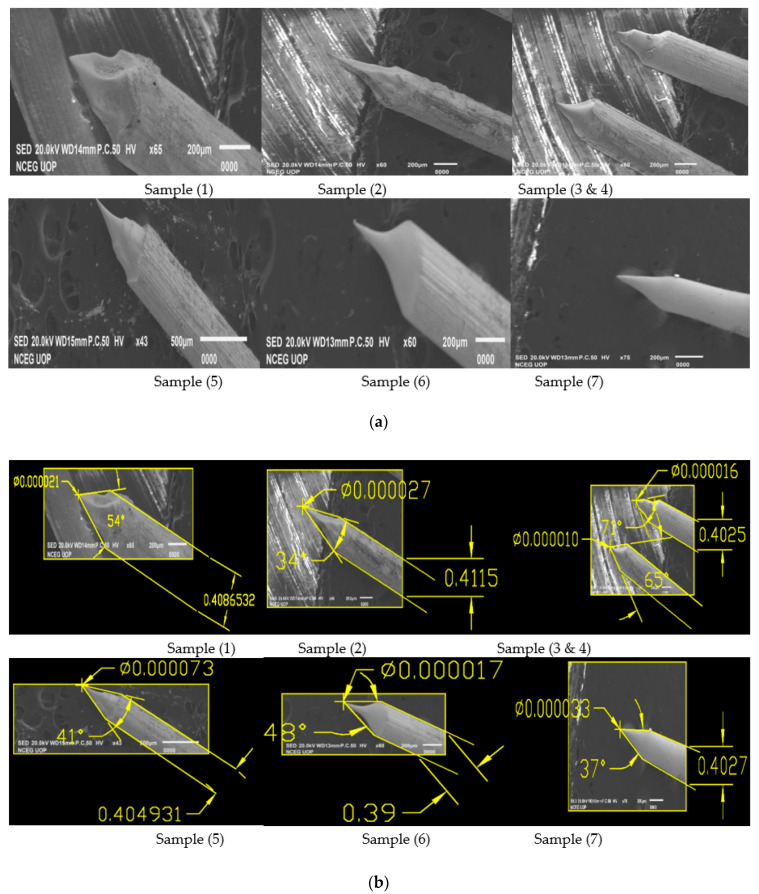
(**a**) SEM Images showing Sample 3 optimized etching parameters for reproducibility having applied voltage of 6.6 V in 2 M NaOH solution; (**b**) SEM Images of tungsten tips showing Sample 3 cone angle and tip diameter measured using AutoCAD Software.

**Figure 3:**
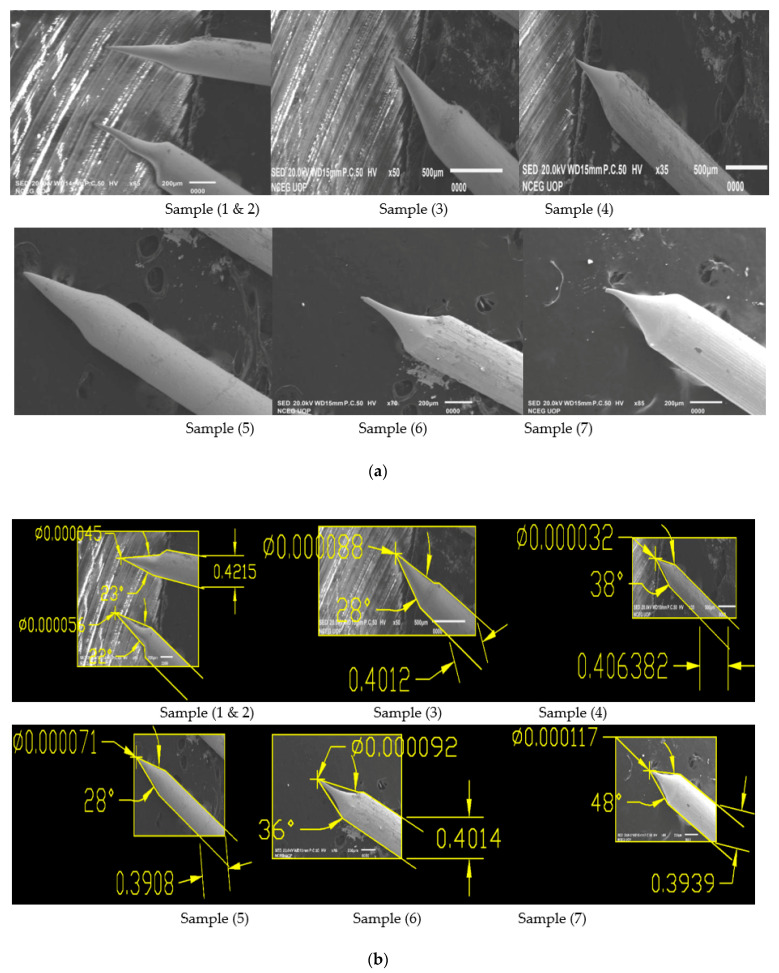
(**a**) SEM Images showing Sample 9 having applied voltage of 12.5 V in 2M NaOH solution; (**b**) SEM Images of tungsten tips showing sample 9 cone angle and tip diameter measured using AutoCAD Software.

From the above finding, the results shown in Table 1 and Figure 1b for produced tips in 2 molar NaOH solution, Sample 3 and Sample 9 of the above tips have the best cone angle and tip diameter, so we have prepared these two samples and checked their sharpness and cone angle, which is one of our objectives [13]. For both samples, we have performed 14 experiments in 2 molar solution keeping the voltage of samples 3 and 9 constant and taken samples. Experimental results for sharp tips produced are shown below in Table 2 while SEM images are shown in Figure 2 and Figure 3.

It can be seen from the results shown in Table 1 that for the original Sample 3 cone angle obtained is 31° and the tip diameter is 88 nm. While for the investigation, we performed 7 experiments on a constant voltage of Sample 3, which is 6.6 V as shown in Table 2 and obtained larger cone angle tips, less tip diameter and approximate etching time than original Sample 3. Best cone angle is 65° on which tip diameter is 10 nm and etching time is 10 min. Similarly, we repeated experimental work for Sample 9 on a constant voltage of 12.5 V for which best cone angle is 38° on which tip diameter is 32 nm and etching time is 5 min.

### 3.2. Meniscus Etching Results

**Figure 4:**
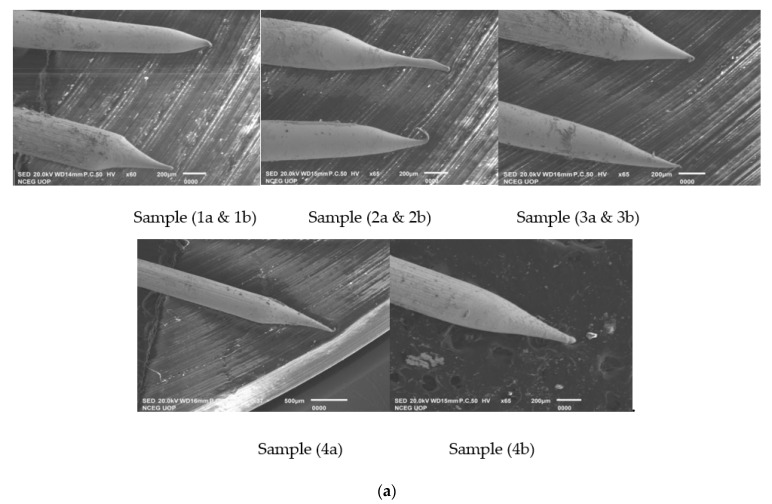

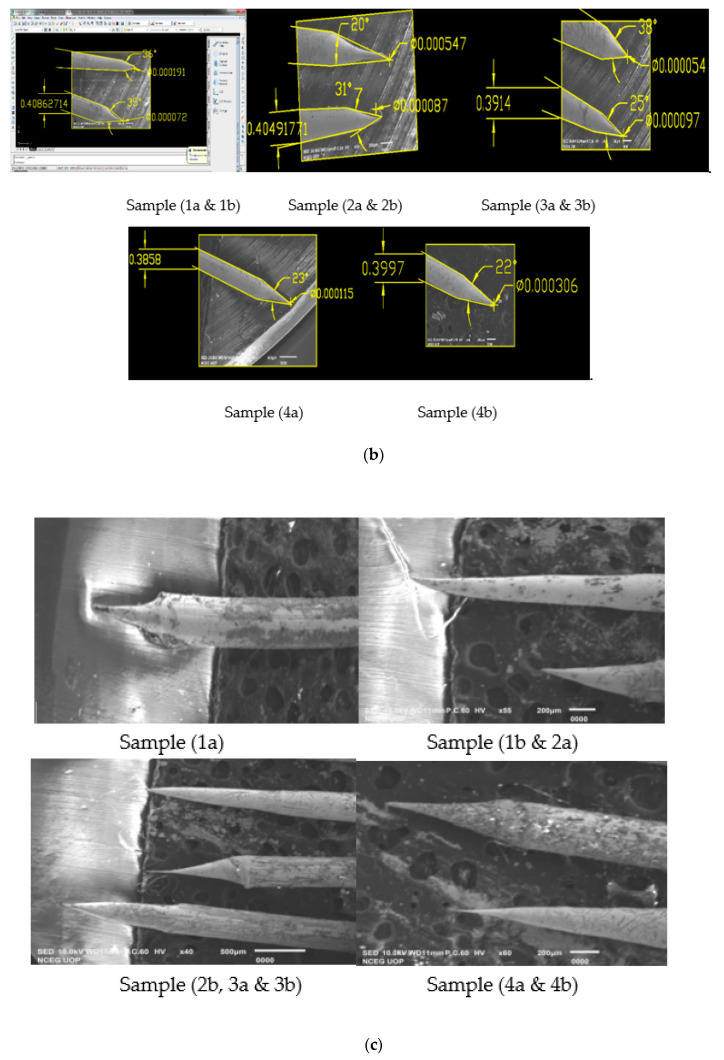

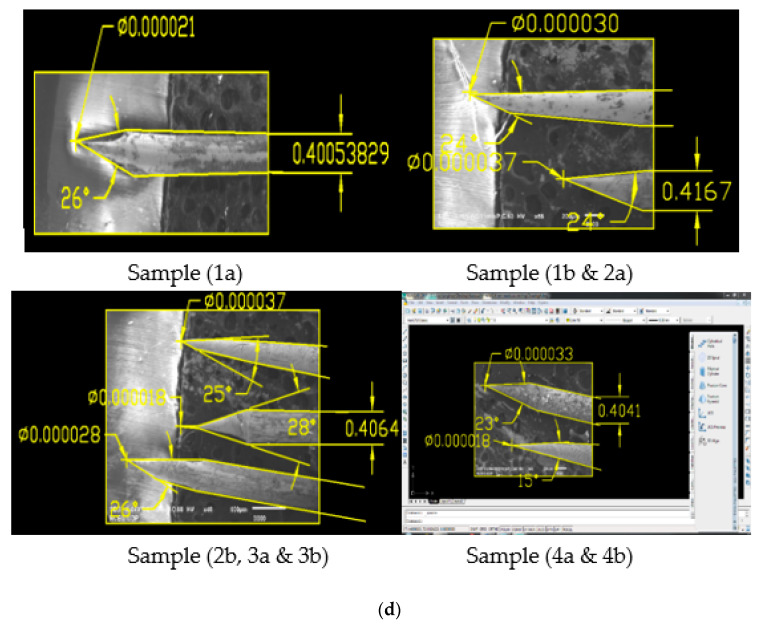
(**a**) SEM Images of 0.4 mm tungsten tips prepared on Meniscus etching in 2 Molar NaOH solution (16 g/200 mL); (**b**) SEM Images of 0.4 mm tungsten tips in 2 molar NaOH solution showing cone angle and tip diameter measured using AutoCAD Software; (**c**) SEM Images of 0.4 mm tungsten tips prepared on Meniscus etching in 4 Molar NaOH solution (32 g/200 mL); (**d**) SEM Images of 0.4 mm tungsten tips in 4 molar NaOH solution showing cone angle and tip diameter measured using AutoCAD Software.

On meniscus etching, 0.4 mm equivalent to 0.016-inch tungsten wire is passed from a ring like structure, that is, a sheet of metal having a small hole containing 2 and 4 molar NaOH solution. We have performed eight (08) experiments on meniscus etching for 0.4 mm tungsten wire in both 2 and 4 molar NaOH solutions. Experimental results for produced tips and SEM images are shown below in Table 3 and Figure 4, respectively.

**Figure 5:**
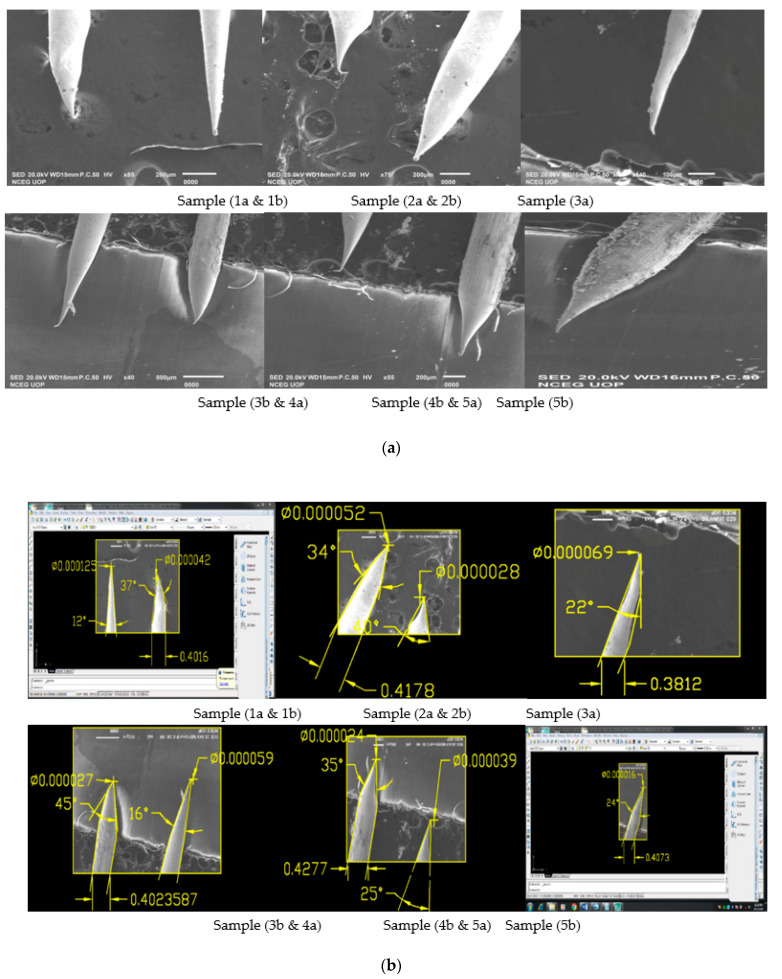
(**a**) SEM Images showing Sample 1a having applied voltage of 6.6 V in 2 M NaOH solution; (**b**) SEM Images of 0.4 mm tungsten tips showing Sample 1a cone angle and tip diameter measured using AutoCAD Software.

**Figure 6:**
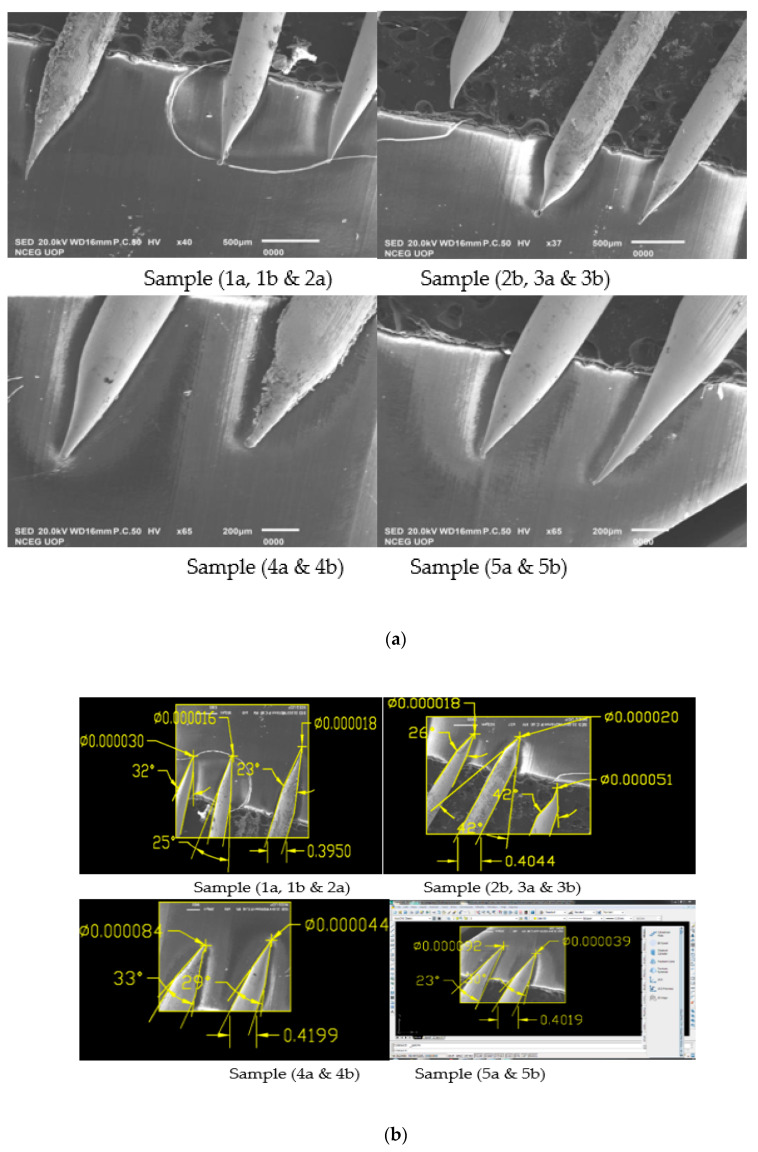
(**a**) SEM Images showing Sample 3b having applied voltage of 8.5 V in 2 M NaOH solution; (**b**) SEM Images of 0.4 mm tungsten tips showing Sample 3b cone angle and tip diameter measured using AutoCAD Software.

Looking to the above results shown in Table 3 and Figure 4b for the produced tips in 2 molar solution, Sample 1a and Sample 3b have the best cone angle and tip diameter, so we have prepared these two samples and checked for their optimum results [13]. For both samples, we have performed 10 experiments in 2 molar solution keeping the voltage of Sample 1a and 3b constant and have taken samples. Experimental results for the reproduced tips are shown below in Table 4 while SEM images are shown in Figure 5 and Figure 6.

It can be seen from the results shown in Table 3 that for the original sample 1a cone angle obtained is 35°, tip diameter is 72 nm and etching time is 9 min. While for process optimization, we performed 5 experiments on constant voltage of sample 1, which is 6.6 V as shown in above Table 4 and obtained larger cone angle tips, less tip diameter and approximate etching time than original sample. Similarly, we repeated experimental work for sample 3b on a constant voltage of 8.5 V for which the best cone angle is 42° greater than the original sample 3, tip diameter is 20 nm less than the original sample 3 and etching time is 5 min as shown in Table 4.

### 3.3. DC Etching Results

**Figure 7:**
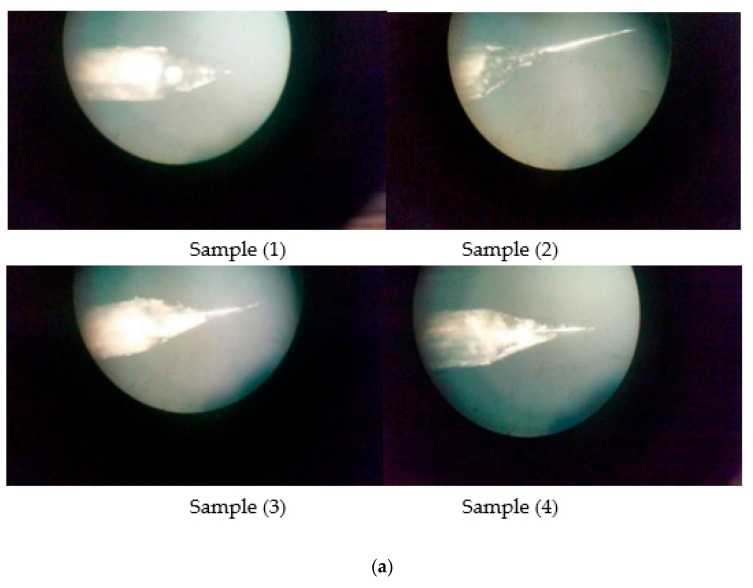

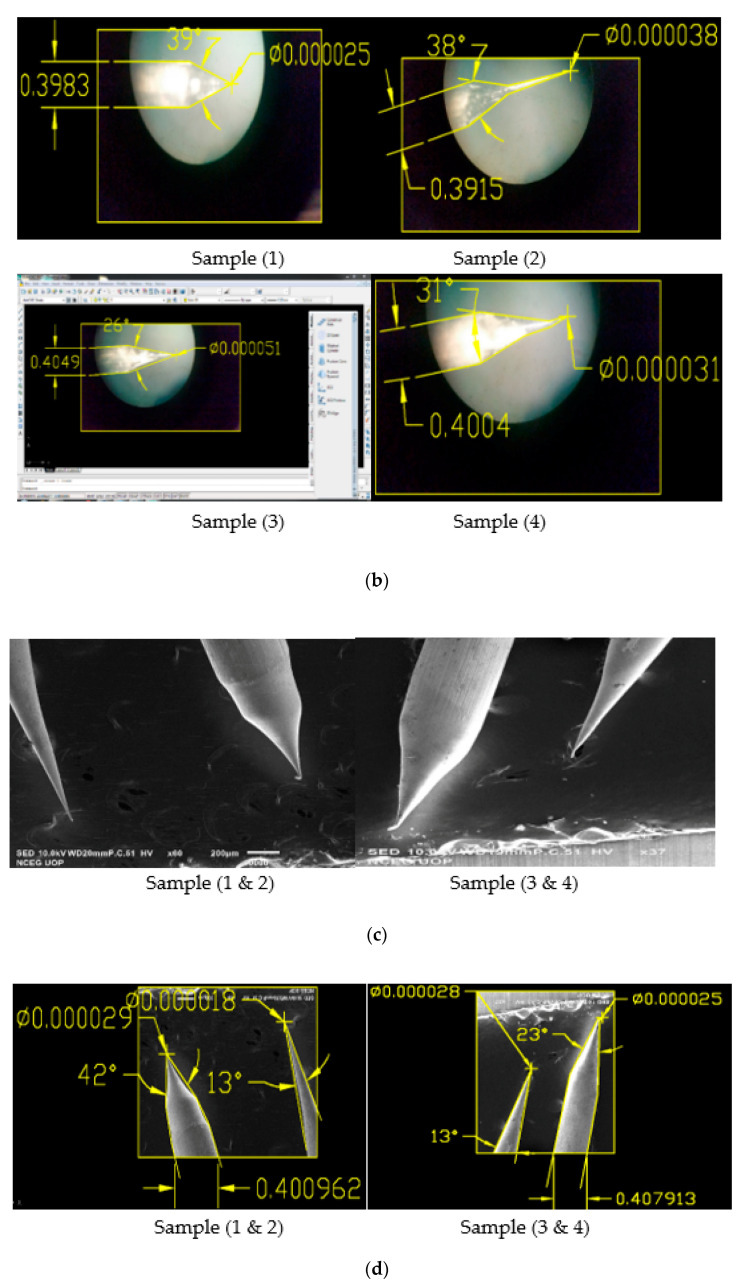
(**a**) OM Images of 0.4 mm tungsten tips prepared on DC etching in 2 Molar NaOH solution (16 g/200 mL); (**b**) OM Images of 0.4 mm tungsten tips showing cone angle and tip diameter measured using AutoCAD Software; (**c**) SEM Images of 0.4 mm tungsten tips prepared on DC etching in 4 Molar NaOH solution (32 g/200 mL); (**d**) SEM Images of 0.4 mm tungsten tips showing cone angle and tip diameter measured using AutoCAD Software.

Here on DC etching we performed 4 experiments for 0.4 mm tungsten wire in 2 and 4 molar NaOH solution using electrochemical etching setup and taken samples. Experimental results and OM images for produced tips are given below in Table 5 and Figure 7.

**Figure 8:**
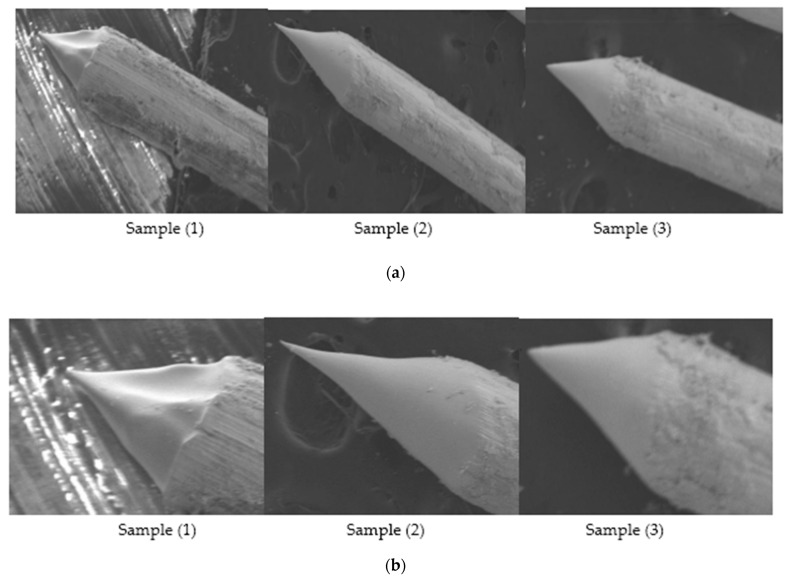

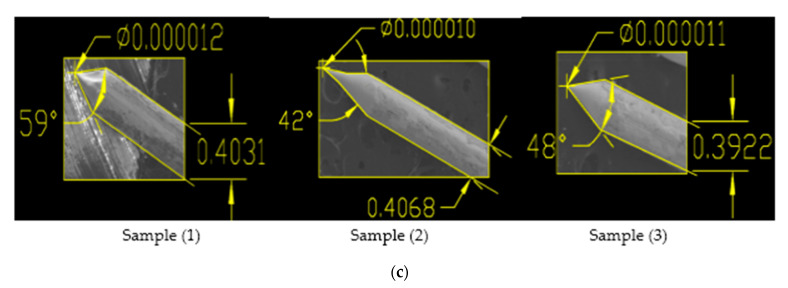
(**a**) SEM Images showing Sample 1 having applied voltage of 6.6 V in 2 M NaOH solution; (**b**) Zoomed-in-View of Sample 1, 2 and 3; (**c**) SEM Images of 0.4 mm tungsten tips showing cone angle and tip diameter measured using AutoCAD Software.

**Figure 9:**
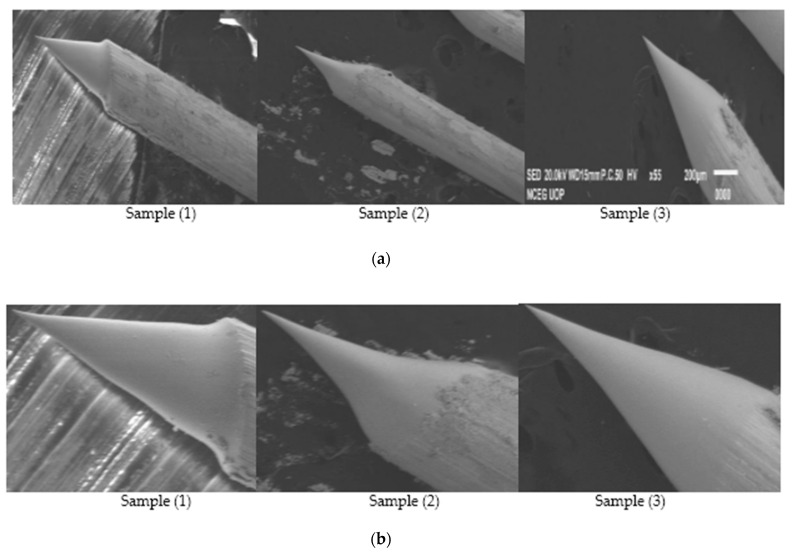

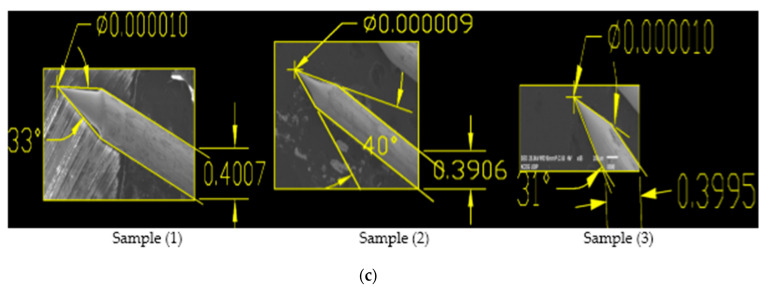
(**a**) SEM Images showing Sample 4 having applied voltage of 12.5 V in 2 M NaOH solution; (**b**) Zoomed-in-View of Sample 1, 2 and 3; (**c**) SEM Images of 0.4 mm tungsten tips showing cone angle and tip diameter measured using AutoCAD Software.

As shown in Table 5 and Figure 7b; Sample 1 and Sample 4 of the above tips produced the best results for both cone angle and tip diameter than the other two tips, so we have prepared these two samples and checked for their diameter and cone angle [13]. Experimental results and SEM images for the produced tips are shown below in Table 6 and Figure 8 and Figure 9, respectively.

From the results shown in Table 5, it is clear that for original sample 1, the cone angle is 39°, the tip diameter is 25 nm and the etching time is 9 min, while for optimization we performed 3 experiments on a constant voltage of 6.6 V and obtained 3 samples each having a larger cone angle, less tip diameter and approximate etching time than original sample as shown in Table 6. For repeated sample 1, the best cone angle is 59°, tip diameter from 10–12 nm and etching time from 6–8 min are obtained. Similarly, we repeated experimental work for sample 4 on constant voltage of 12.5 V for which best cone angle is 40°, tip diameter is 9 nm and etching time is 4 min as shown in above Table 6. Moreover looking to the above Table 2, Table 4 and Table 6, comparing results to the original samples, it is clear that our technique is optimized and can produce the required Tungsten sharp probes by keeping the reference voltage and experimental condition constant.

### 3.4. EDX Analysis

**Figure 10:**
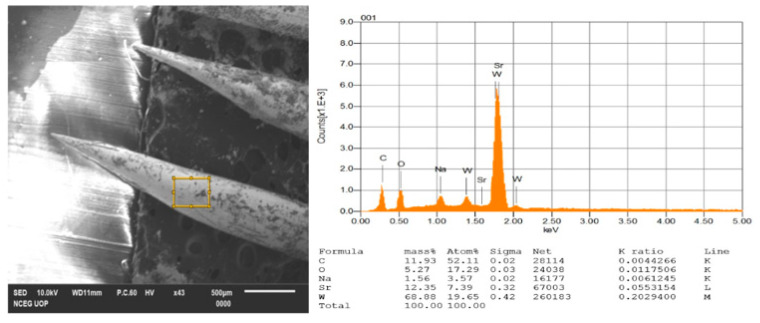
Energy Dispersive X-ray (EDX) results for a heavily contaminated tungsten tip.

**Figure 11:**
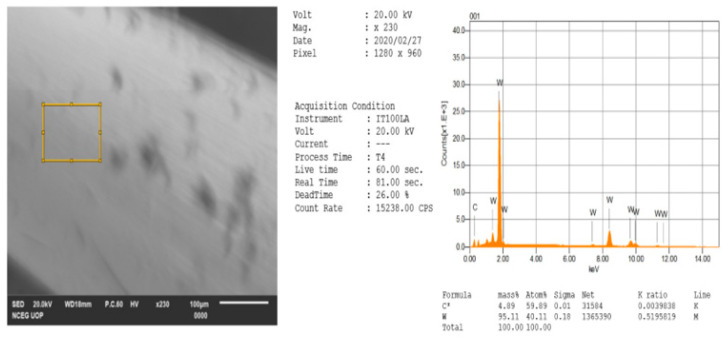
EDX results for a tungsten tip after removal of contamination.

Using an Energy Dispersive X-ray (EDX) study, we confirmed the chemical composition of the produced tips after conducting experiments on tungsten wire, and also analyzed contamination/oxidation layers [22]. As is clear from Figure 10 and Figure 11, our original sample, that is, 0.4 mm tungsten wire, is 99.9% pure tungsten wire.

Instead of it, we have performed these two EDX analyses for tungsten tips after electrochemical etching whose results are shown below in Figure 10 and Figure 11. We have performed first EDX analysis for tips after the preparation of electrochemical etching process without rinsing and cleaning with distilled water, iso-propanol and concentrated HF (Hydro fluoric acid) and so forth. So, due to a higher degree of oxidation process and impurities, some of the other particles like Na, O and C and so forth react with tungsten to form a tungsten oxide layer as clear from the literature review and is also shown here. The oxidation layer is very thin and hard and needs to be removed for accurate analysis and resolution of the tips. From the EDX analysis performed for our specific tip, the chemical composition of contaminations present consists of Carbon (C), Oxygen (O), Sodium (Na), Strontium (St) and tungsten (W) whose actual percentages are shown in Figure 10.

After the result of the first EDX analysis, which shows contaminations and oxidation, so we have performed a second EDX analysis for tungsten tip rinsed with distilled water, iso-propanol and lastly in concentrated Hydro fluoric (HF) acid, which have removed some of the contaminations and tungsten oxide layer present. Moreover, tungsten tips need to be stored in a vacuum chamber after rinsing for better results. The results of the second EDX analysis are shown below in Figure 11, which consists of 95.11 percent of tungsten and 4.89 percent of carbon, the rest of the oxide layers have been removed.

**Figure 12:**
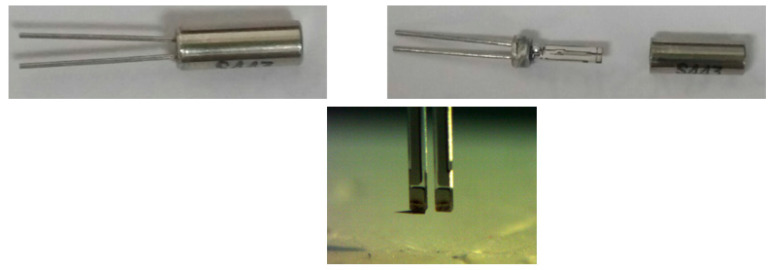
Canister removal and tungsten tip attachment to QTF.

**Figure 13:**
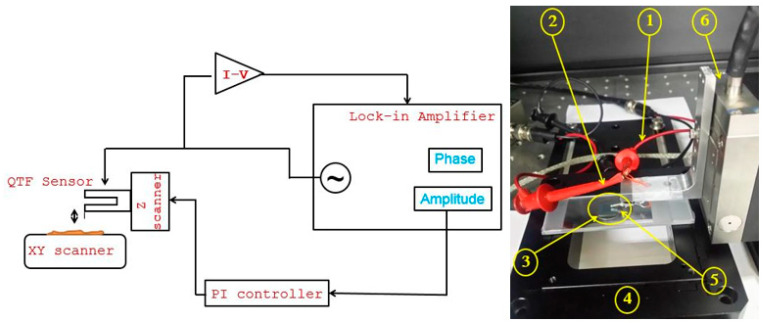
Schematic and Proof-of-concept experimental setup of Amplitude modulation imaging mode.

**Figure 14:**
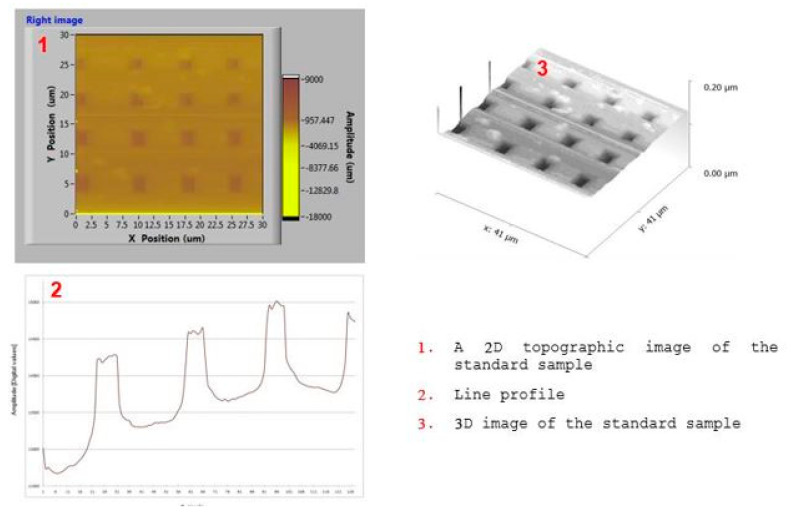
Non-contact atomic force microscopy (AFM) imaging of calibration sample.

The functionality of the manufactured tungsten tips as a QTF sensor is evaluated by implementing Amplitude modulated AFM in its basic configuration. The tungsten tip is attached to quartz tuning fork (QTF) as shown in Figure 12 and then tuned near resonance frequency in order to check its quality factor. The QTF Sensor is excited at a constant frequency and amplitude. The amplitude change and the phase shift reflects the interaction between the tip and sample due to wonder walls forces that are measured by a lock-in amplifier as shown in Figure 13. Consequently, in order to highlight the topographic capabilities of the developed QTF sensor, topographic images of a standard calibration sample are shown in Figure 14.

Input signal ($V_{Osc}$) 2. Output signal ($I_{Sig}$) 3. Standard sample 4. X-Y scanner 5. QTF sensor 6. Z-scanner

As the tip should be mounted at the end of a prong of QTF, a low mass of tip is important not to reduce the Q-value of the tuning fork, and that is the reason why a thin wire was chosen and etched to a conical shape accordingly.

## 4. Conclusions

SPM tips with larger cone angles are able to produce atomically resolved images of the selected specimen due to their greater mechanical stability and strength. There are several electrochemical etching techniques for the production of SPM tips that allows to produce larger cone angles and high-quality tips. In general, tips with a cone angle of ~15° or greater gave excellent SPM images and a cone angle of ~60° would be optimal for both stability and sharpness. Therefore, as a step towards this goal, in this research, we have selected 0.4 mm thick tungsten wire for the production of tips by using three types of electrochemical etching techniques. We used electrochemical etching techniques, that is, AC Etching, Meniscus Etching and DC Etching methods to produce tips. Moreover, effect of voltage and concentration of solution on tip diameter, cone angle and etching time is also investigated. A high concentration of NaOH and applied voltage is seen to produce hydrogen bubbles on the cathode side, which disturbs the solution and tungsten tip. So, NaOH concentration is kept in a suitable amount to minimize chances of bubble production, which will affect tip sharpness. As the concentration of solution and applied voltage increases, the reaction rate becomes faster and it takes less time to complete the etching. Etching time is found to have an inverse relation to applied voltage and concentration of solution. Moreover, as concentration of solution increases, sharp tips with a long taper length and fewer cone angles are produced, which are not desirable for SPM. Tip sharpness also depends on the position of cathode to specimen, that is, anode, as cathode comes closer to anode, the reaction rate increases and as a result sharp tips are produced. It is observed that increasing space between cathode and anode does not affect the results for both AC and DC etching but the etching time increases while, for the same setup, meniscus etching produces blunt tips with a larger taper length having less cone angle due to the continuous falling of etching solution on the specimen. Similarly, we observed that a dipped length of 2.5–3 mm gave optimum results for both cone angle and tip sharpness.

A successful attempt is made for optimizing the etching parameters of some typical samples produced with the mentioned three methods is investigated where we obtained 78%, 60% and 100% sharp tips from conventional AC, meniscus and DC etching, respectively. Moreover, we concluded that the DC etching technique, for the production of 0.4 mm tungsten tips, is very appropriate.

In Figure 10 and Figure 11, it is shown that the tips need to be cleaned and rinsed with antioxidants and distilled water after electrochemical etching, which will remove the oxidation layer. Moreover; to limit oxidation and contamination layers, tips need to be stored in a vacuum chamber or dry cabinet. Finally, for a tip diameter of less than 50 nm and a cone angle of greater than 20°, ~40%, ~70% and 90% success rate is obtained for AC, meniscus and DC etching respectively.

The prepared tungsten tips are used for quartz tuning fork based AFM and the topographic images of the calibration sample are obtained.

## Figures and Tables

**Table 1:** Experimental results for 0.4 mm tips showing tip diameter, etching time and Cone Angle produced in 2 M and 4 M NaOH solution on AC Etching.

S. No	Voltage (V)	Tip Diameter (nm)		Etching Time (min)		Cone Angle (deg)	
		2 M	4 M	2 M	4 M	2 M	4 M
1	3.8	94	-	18	-	28°	-
2	4.9	-	119	13	4	35°	20°
3	6.6	88	58	9	4	31°	14°
4	7.3	91	41	7	3	37°	14°
5	8.5	-	-	8	-	37°	-
6	9.2	-	13	7	2	34°	19°
7	10.5	-	-	7	-	-	-
8	11.2	-	-	6	-	-	-
9	12.5	63	-	3	-	23°	-

**Table 2:** Experimental results for 0.4 mm tips showing optimized results of Sample 3 and 9 in 2 M NaOH solution on AC Etching.

S. No	Repeated Sample 3				Repeated Sample 9			
	Voltage (V)	Cone Angle (deg)	Tip Diameter (nm)	Etching Time (min)	Voltage (V)	Cone Angle (deg)	Tip Diameter (nm)	Etching Time (min)
1	6.6	54°	21	12	12.5	22°	56	4
2	6.6	34°	27	9	12.5	23°	45	3
3	6.6	65°	10	10	12.5	28°	88	4
4	6.6	71°	16	12	12.5	38°	32	5
5	6.6	41°	73	13	12.5	28°	71	3
6	6.6	48°	17	10	12.5	36°	92	2
7	6.6	37°	33	13	12.5	48°	117	5

**Table 3:** Experimental results for 0.4 mm tips showing tip diameter, etching time and cone angle produced in 2 M and 4 M NaOH solution on Meniscus Etching.

S. No	Voltage (V)	Tip Diameter (nm)		Etching Time (min)		Cone Angle (deg)	
		2 M	4 M	2 M	4 M	2 M	4 M
1	6.6	72	21	9	2	35°	26°
		-	37			36°	24°
2	7.3	87	30	7	2	31°	24°
		-	28			20°	26°
3	8.5	97	18	6	1	25°	28°
		54	37			38°	25°
4	9.2	115	18	5	1	23°	15°
		-	33			22°	23°

**Table 4:** Experimental results for 0.4 mm tips showing optimal results of Sample 1a and 3b in 2 M NaOH solution on Meniscus Etching.

S. No	Repeated Sample 1				Repeated Sample 3			
	Voltage (V)	Cone Angle (deg)	Tip Diameter (nm)	Etching Time (min)	Voltage (V)	Cone Angle (deg)	Tip Diameter (nm)	Etching Time (min)
1	6.6	37°	42	11	8.5	23°	18	4
		12°	-			25°	16	
2	6.6	40°	28	7	8.5	32°	30	6
		34°	52			42°	51	
3	6.6	45°	27	5	8.5	42°	20	5
		22°	69			26°	18	
4	6.6	35°	24	5	8.5	29°	44	4
		16°	59			33°	84	
5	6.6	25°	39	6	8.5	30°	39	4
		24°	16			23°	92	

**Table 5:** Experimental results for 0.4 mm tips showing tip diameter, Etching time and Cone Angle produced in 2 M NaOH solution on DC Etching.

S. No	Voltage (V)	Tip Diameter (nm)		Etching Time (min)		Cone Angle (deg)	
		2 M	4 M	2 M	4 M	2 M	4 M
1	6.6	25	18	9	8	39°	13°
2	8.5	38	29	8	6	38°	41°
3	10.5	51	25	6	4	26°	23°
4	12.5	31	28	5	3	31°	13°

**Table 6:** Experimental results for 0.4 mm tips showing optimal results of Sample 1 and 4 produced in 2 M NaOH solution on DC Etching.

S. No	Repeated Sample 1				Repeated Sample 4			
	Voltage (V)	Cone Angle (deg)	Tip Diameter (nm)	Etching Time (min)	Voltage (V)	Cone Angle (deg)	Tip Diameter (nm)	Etching Time (min)
1	6.6	59°	12	8	12.5	33°	10	5
2	6.6	42°	10	6	12.5	40°	9	4
3	6.6	48°	11	8	12.5	31°	10	5
